# Rho GTPase gene expression and breast cancer risk: a Mendelian randomization analysis

**DOI:** 10.1038/s41598-022-05549-5

**Published:** 2022-01-27

**Authors:** Nabila Kazmi, Tim Robinson, Jie Zheng, Siddhartha Kar, Richard M. Martin, Anne J. Ridley

**Affiliations:** 1 MRC Integrative Epidemiology Unit (IEU), Bristol Medical School, University of Bristol, Bristol, BS8 2BN UK; 2 Population Health Sciences, Bristol Medical School, University of Bristol, Bristol, BS8 2BN UK; 3 National Institute for Health Research (NIHR) Bristol Biomedical Research Centre, University Hospitals NHS Trust and University of Bristol, Bristol, UK; 4 School of Cellular and Molecular Medicine, University of Bristol, Bristol, UK

**Keywords:** Cancer, Genetics

## Abstract

The Rho GTPase family consists of 20 genes encoding intracellular signalling proteins that influence cytoskeletal dynamics, cell migration and cell cycle progression. They are implicated in breast cancer progression but their role in breast cancer aetiology is unknown. As aberrant Rho GTPase activity could be associated with breast cancer, we aimed to determine the potential for a causal role of Rho GTPase gene expression in breast cancer risk, using two-sample Mendelian randomization (MR). MR was undertaken in 122,977 breast cancer cases and 105,974 controls, including 69,501 estrogen receptor positive (ER+) cases and 105,974 controls, and 21,468 ER negative (ER−) cases and 105,974 controls. Single nucleotide polymorphisms (SNPs) underlying expression quantitative trait loci (eQTLs) obtained from normal breast tissue, breast cancer tissue and blood were used as genetic instruments for Rho GTPase expression. As a sensitivity analysis, we undertook co-localisation to examine whether findings reflected shared causal variants or genomic confounding. We identified genetic instruments for 14 of the 20 human Rho GTPases. Using eQTLs obtained from normal breast tissue and normal blood, we identified evidence of a causal role of *RHOD* in overall and ER+ breast cancers (overall breast cancer: odds ratio (OR) per standard deviation (SD) increase in expression level 1.06; (95% confidence interval (CI) 1.03, 1.09; P = 5.65 × 10^–5^) and OR 1.22 (95% CI 1.11, 1.35; P = 5.22 × 10^–5^) in normal breast tissue and blood respectively). There was a consistent direction of association for ER− breast cancer, although the effect-estimate was imprecisely estimated. Using eQTLs from breast cancer tissue and normal blood there was some evidence that *CDC42* was negatively associated with overall and ER + breast cancer risk. The evidence from colocalization analyses strongly supported our MR results particularly for *RHOD*. Our study suggests a potential causal role of increased *RHOD* gene expression, and, although the evidence is weaker, a potential protective role for *CDC42* gene expression, in overall and ER+ breast cancers. These finding warrant validation in independent samples and further biological investigation to assess whether they may be suitable targets for drug targeting.

## Introduction

**Figure 1:**
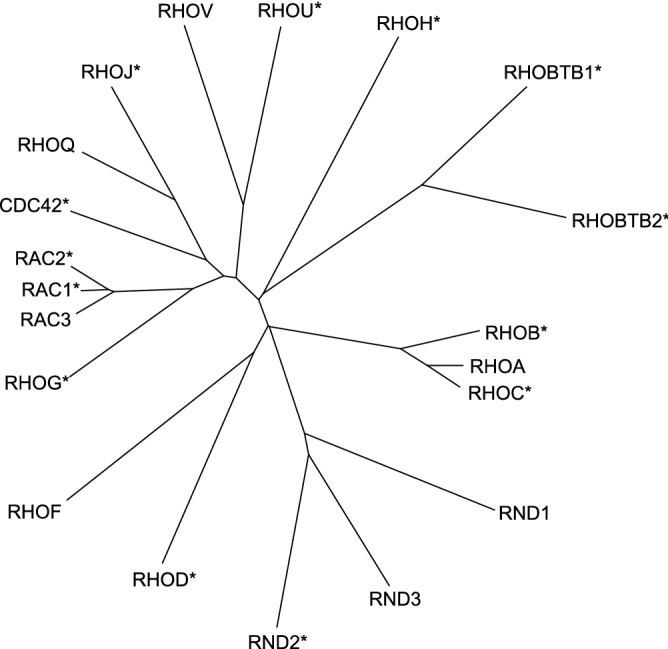
Twenty human Rho GTPase family members. A ClustlW alignment using the amino acid sequences of the 20 human Rho GTPases was used to generate the phylogenetic tree. *, 14 genes able to be analysed by MR (see Table 1).

The Rho family of GTPases are key molecular regulators of actin and microtubule cytoskeletal dynamics that influence oncogenic processes such as cellular adhesion, migration, survival and cell cycle progression^1^. There are 20 Rho family genes in humans^2^ (Fig. 1) and several Rho GTPases, and their associated signalling pathways, have been implicated in biological processes involved in cancer initiation and progression^3,4^. For example, increased expression of RhoA results in malignant transformation of mouse fibroblasts, stimulating carcinogenesis in a mouse model, and loss of function of RhoB increases chemically-induced carcinogenesis in an in vivo skin cancer model^5,6^. In breast cancer, multiple in vitro and in vivo experimental studies, across all breast cancer subtypes, have implicated a role for aberrant Rho GTPase activity in aggressive biological phenotypes^1,3,4,7^. However, a causal role of abberant Rho GTPase signalling in the risk of developing breast cancer in humans is less clear.

**Table 1:** Results of power calculation for the main analysis (using top eQTL).

Gene	Resource	Tissue	R^2^	F-statistic	Power (overall BC)	Power (ER+ BC)	Power (ER− BC)
*RHOD*	GTEx	Breast mammary	27.28	93.42	> 99	> 99	> 99
*RHOD*	eQTLGen	Blood	0.35	107.03	> 99	59.63	30.06
*CDC42*	eQTLGen	Blood	3.41	1118.52	> 99	> 99	> 99
*CDC42*	TCGA	Breast cancer	0.31	14.97	67.57	54.56	27.19
*RHOBTB1*	GTEx	Breast mammary	20.76	65.24	> 99	> 99	> 99
*RHOBTB1*	eQTLGen	Blood	1.85	582.89	> 99	> 99	91.20
*RHOBTB2*	eQTLGen	Blood	0.49	154.52	86.01	74.25	39.85
*RHOU*	eQTLGen	Blood	2.95	803.91	> 99	> 99	98.70
*RHOU*	TCGA	Breast cancer	0.34	16.00	70.45	57.32	28.72
*RHOQ*	eQTLGen	Blood	1.07	257.98	> 99	97.16	71.24
*RHOQ*	TCGA	Breast cancer	0.44	21.29	82.09	69.57	36.40
*RHOH*	eQTLGen	Blood	0.31	97.50	67.34	54.34	27.07
*RHOG*	eQTLGen	Blood	0.65	206.04	93.78	85.14	49.93
*RHOG*	TCGA	Breast cancer	0.35	16.90	72.78	59.63	30.06
*RHOC*	eQTLGen	Blood	1.90	609.56	> 99	> 99	91.90
*RHOB*	eQTLGen	Blood	0.59	188.24	91.67	81.88	46.50
*RHOB*	TCGA	Breast cancer	0.27	12.94	61.28	48.77	24.13
*RHOJ*	TCGA	Breast cancer	0.54	26.11	89.01	78.13	43.04
*RAC1*	eQTLGen	Blood	2.03	546.86	> 99	> 99	93.46
*RAC1*	TCGA	Breast cancer	0.28	13.67	63.65	50.91	25.24
*RAC2*	eQTLGen	Blood	2.65	863.39	> 99	> 99	97.77
*RAC2*	TCGA	Breast cancer	0.34	16.44	71.62	58.47	29.38
*RND2*	TCGA	Breast cancer	0.78	38.02	97.00	90.96	57.58

BC = breast cancer; R^2^ represents the variance explained in the expression level of the gene by the instrument; F-statistic indicates strength of the instrument used for each gene expression (a strong instrument is sometimes defined as an F-statistic > 10); and the Power represents the power to detect an odds ratio of 1.2 (or 0.80) for an association of the expression of gene expression with breast cancer at an alpha-level of 0.05, given the values in the R^2^ column and the number of breast cancer cases and controls.

Mendelian randomization (MR) uses germline genetic variants as instruments (“proxies”) to generate evidence for an association of potentially modifiable extrinsic risk factors and intrinsic metabolic processes on disease outcomes^8,9^. The aim of using germline genetic instruments is to minimise confounding and reverse causation, as germline genotype is assigned at random at conception and fixed thereafter. These properties can make MR-derived effect-estimates independent of confounding by future lifestyle or environmental factors, and less likely to be affected by reverse causation, provided the following three assumptions are met: (i) robust association of the genetic instrument with the exposure of interest; (ii) no confounding of the instrument-outcome relationship; and (iii) lack of an alternative pathway through which an instrument influences the outcome except through the exposure^8^. The feasiblity, precision and statistical power of MR analysis can be increased by using a “two-sample MR” framework in which summary genetic association data from independent samples representing firstly, genetic variant-exposure and secondly, genetic variant-outcome associations are combined in order to assess causal effects^10^.

The aim of our study was to assess whether a potential association exists between the expression of genes encoding Rho GTPases and risk of overall, estrogen receptor-positive (ER+) and estrogen receptor-negative (ER−) breast cancer.

## Methods

### Study population

Summary data were obtained from a genome-wide association study (GWAS) of 122,977 overall breast cancer cases (including 69,501 ER+ and 21,468 ER− breast cancer cases) and 105,974 controls of European ancestry from the Breast Cancer Association Consortium (BCAC)^11^. All participating studies in BCAC were approved by their appropriate ethics review board and all subjects provided informed consent and all the methods were performed in accordance with the relevant guidelines and regulations. Full details of the 78 studies that contributed data to this BCAC analysis and their ethical approval committees are listed in^11^.

In studies that participated in BCAC, the ER status provided by each individual study was usually from either; (1) clinical records, (2) immunohistochemistry (IHC) whole sections, (3) IHC stain of Tissue Micro Arrays (TMAs), (4) derived from Ariol data of TMAs (a high throughput automated imaging system) (5) derived from semi-quantitative IHC stain data (not defined whether from whole sections or TMAs), and (6) other (not defined). Sometimes, the source is unknown.

**Figure 2:**
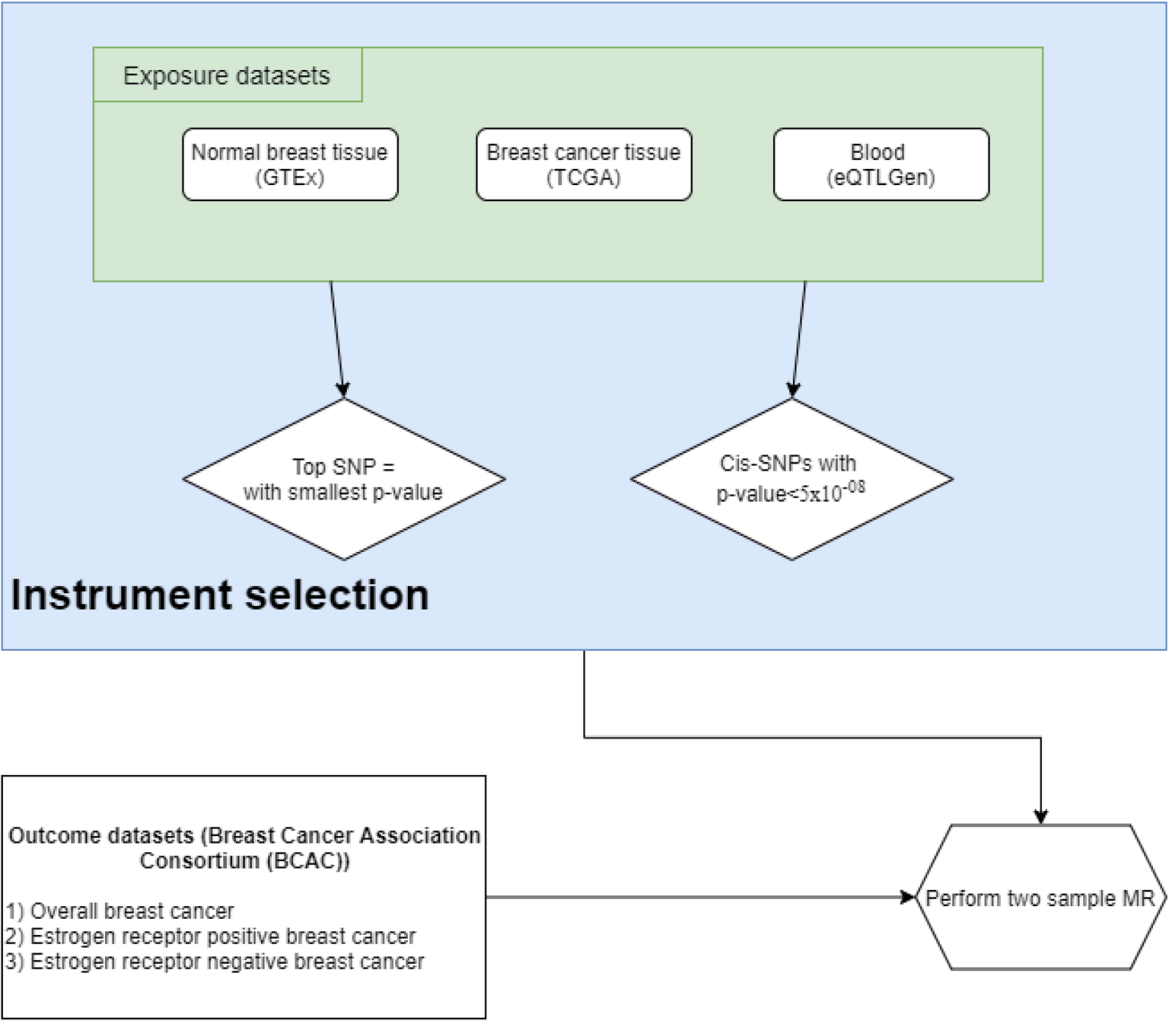
A diagramatic representation of the datasets included and the methodology is shown as a flow diagram.

A diagrammatic representation of the included datasets and the methodology is shown as a flow diagram (Fig. 2).

### Instrument construction

To generate genetic instruments to proxy for Rho GTPase gene expression, we performed a multi-step approach. First, single nucleotide polymorphisms (SNPs) marking expression quantitative trait loci (eQTLs) underlying expression of the genes encoding 20 Rho GTPases were obtained from normal breast tissue, breast cancer tissue and blood from patients without breast cancer. We obtained normal breast tissue specific eQTLs by searching for the expression of each gene in the Genotype-Tissue Expression (GTEx) project (v8) (https://gtexportal.org/home/)^12^ and selected the top SNP associated with the gene expression (defined by the smallest *P*-value). Second, we extracted eQTLs (the top SNP; smallest *P*-value) from the eQTLGen consortium (https://www.eqtlgen.org/) which has performed cis- and trans-eQTL analysis in blood from 31,684 individuals^13^ of largely European ancestry. The consortium defined the cis-eQTLs as every SNP-gene combination with a distance < 1 Mb from the center of the gene and were tested in at least 2 cohorts. Third, we obtained breast cancer tissue eQTLs from The Cancer Genome Atlas (TCGA) (https://albertlab.shinyapps.io/tcga_eqtl/) which has systematically performed eQTL analyses across 24 human cancer types^14^. Finally, we selected cis-SNPs (within 1 Mb of the target gene) that were associated with gene expression level at a *P*-value threshold of P < 5 × 10^–08^ based on summary data available on the three platforms. To retain only independent SNPs, we used linkage disequilibrium [LD] clumping with a threshold of *r*^2^ ≤ 0.01 based on the 1000 Genomes European ancestry reference panel data^15^.

In sensitivity analyses, *TP53* gene expression was used as a control in this study. the genetic instrument to proxy this (rs78378222; a germline variant in *TP53* with ability to influence p53 activity)^16,17^ was only available in the eQTLGen consortium dataset.

### Two-sample MR analysis

We extracted the following information for each selected eQTL—effect allele, other allele, beta coefficient and standard error—and calculated the proportion of variance in gene expression explained by the SNP. We calculated R^2^ and F-statistics to assess for both the strength of the genetic instruments and for weak instrument bias using previously reported methods^18^. Exposure and outcome data were harmonised such that the effect of each SNP on the outcome and exposure was relative to the same allele^19^.

For our primary analyses using the top eQTL, we used the Wald ratio method, equivalent to β_YG/_β_XG_ (where Y = outcome [overall, ER+, and ER− breast cancer], G = germline genetic variant, and X = Rho GTPase gene expression). In secondary analyses, when the genetic instrument consisted of multiple SNPs (‘a multi-allelic instrument’), we used the inverse-variance weighted (IVW) method, which performs an inverse variance weighted meta-analysis of each Wald ratio for each SNP^20^. By default our inverse-variance weighted (IVW) Mendelian randomisation analyses were based on random effects models. If under dispersion in these IVW models was detected, i.e., if the residual standard error of the IVW model was less than 1, the standard errors of the regression coefficients were corrected by dividing by 1 rather the by the residual standard error^21^.

We used summary genetic association data (beta coefficients and standard errors) and conducted colocalisation analysis^22^ to investigate the probability that the genetic associations with both gene expression level and risk of breast cancer shared the same underlying causal variants. Here, we used the SNPs that were located within 1 Mb windows of the eQTL studied in the MR analysis.

For multi-allelic instruments, it was possible to perform sensitivity analyses to assess whether there was any evidence of violations of the MR assumption of no pleiotropic effects. For an instrument made up of ≥ 2 independent SNPs*,* Cochran’s Q was computed to quantify heterogeneity across the individual causal effects of SNPs and for an instrument with ≥ 3 independent SNPs, weighted median^23^, weighted mode^24^ and MR-Egger regression^25^ methods were applied. Violations of the MR ‘no horizontal pleiotropy’ assumption (where a locus influences the outcome through independent pathways separate to the exposure)^26^) were assessed by visual inspection of plots constituting a multi-allelic instrument (funnel^27^, forest, scatter and leave-one-out^19^).

To account for multiple testing, Bonferroni corrections were used to establish *P*-value thresholds for strong evidence (P < 0.004; alpha of 0.05/14 genes) and suggestive evidence (0.004 < P < 0.05) of a causal effect.

All analyses were carried out using the TwoSampleMR and MRInstruments R packages, curated by the latest version of MR-Base (0.5.4)^19^, http://www.mrbase.org. The study was in accordance with relevant guidelines and regulations.

## Results

**Figure 3:**
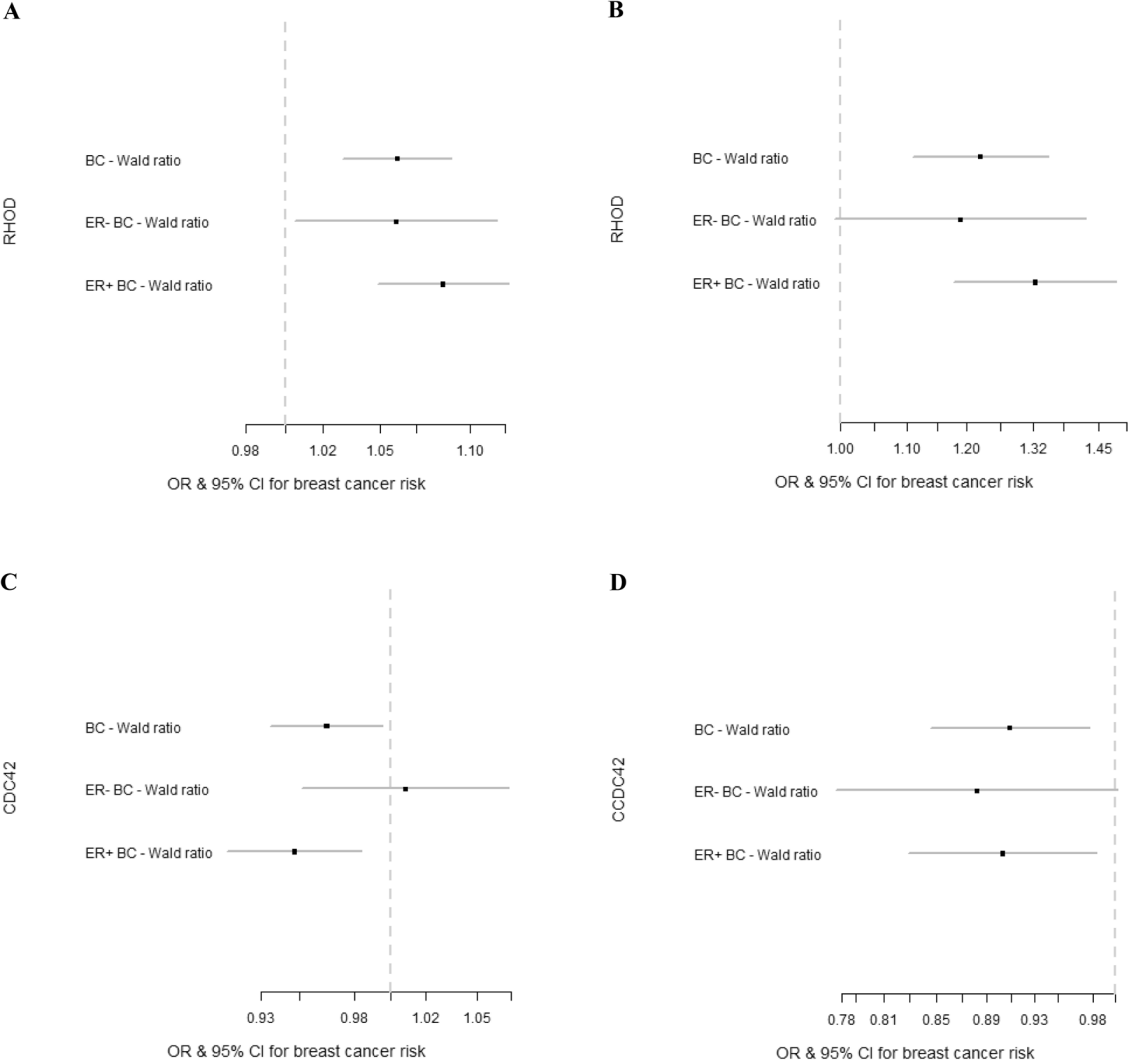
Results of MR analyses performed for overall, ER+ and ER− breast cancer risk for *RHOD* and *CDC42*. The genetic instruments for *RHOD* were obtained from breast mammary tissue (GTEx) (**A**) and from blood (eQTLGen) (**B**) and for *CDC42* were obtained from blood (eQTLGen) (**C**) and from breast cancer tissue (TCGA) (**D**). *BC* breast cancer, *OR* odds ratio, *CI* confidence interval.

Of the 20 genes of the Rho GTPase family, 14 could be analysed using MR as they had at least one strongly associated SNP in at least one of the three databases that were searched (Fig. 1). Of these 14 genes, two showed evidence of an association of an eQTL with breast cancer risk (Table 2 and Fig. 3).

**Table 2:** Mendelian randomisation analyses of the association of *RHOD* and *CDC42* with overall, ER+ and ER− breast cancer risk.

Outcome	Exposure (gene expression level)	Tissue (data source)	MR method	nsnp	OR	LCI	UCI	P-value
Overall breast cancer	*RHOD*	Breast mammary (GTEx)	Wald ratio	1	1.06	1.03	1.09	5.65 × 10^–5^
ER+ breast cancer	*RHOD*	Breast mammary (GTEx)	Wald ratio	1	1.08	1.05	1.12	2.29 × 10^–5^
ER− breast cancer	*RHOD*	Breast mammary (GTEx)	Wald ratio	1	1.06	1.01	1.12	0.03
Overall breast cancer	*RHOD*	Blood (eqtlGen)	Wald ratio	1	1.22	1.11	1.35	5.22 × 10^–5^
ER+ breast cancer	*RHOD*	Blood (eqtlGen)	Wald ratio	1	1.32	1.18	1.49	2.74 × 10^–6^
ER− breast cancer	*RHOD*	Blood (eqtlGen)	Wald ratio	1	1.19	0.99	1.42	0.06
Overall breast cancer	*CDC42*	Breast cancer (TCGA)	Wald ratio	1	0.91	0.84	0.98	0.02
ER+ breast cancer	*CDC42*	Breast cancer (TCGA)	Wald ratio	1	0.90	0.82	0.99	0.03
ER− breast cancer	*CDC42*	Breast cancer (TCGA)	Wald ratio	1	0.88	0.76	1.02	0.09
Overall breast cancer	*CDC42*	Blood (eqtlGen)	Wald ratio	1	0.96	0.93	1.00	0.03
ER+ breast cancer	*CDC42*	Blood (eqtlGen)	Wald ratio	1	0.95	0.91	0.98	0.005
ER− breast cancer	*CDC42*	Blood (eqtlGen)	Wald ratio	1	1.01	0.95	1.07	0.78

*nsnp* number of SNPs used in the analysis, *OR* odds ratio, *LCI* 95% lower confidence interval, *UCI* 95% upper confidence interval, *P-value* P-value for association.

Using eQTLs obtained from both normal breast tissue and blood, increased expression of *ras homolog family member D* (*RHOD)* was positively associated with overall and ER+ breast cancer risk (Overall breast cancer: Odds ratio (OR) per standard deviation (SD) increase in expression level 1.06; (95% confidence interval (CI) 1.03, 1.09; P = 5.65 × 10^–5^) and OR 1.22 (95% CI 1.11, 1.35; P = 5.22 × 10^–5^) in normal breast tissue and blood respectively. ER+ breast cancer: OR per SD increase in expression level was 1.08 (95% CI 1.05, 1.12; P = 2.29 × 10^–5^) and 1.32 (95% CI 1.18, 1.49; P = 2.74 × 10^–6^) in normal breast tissue and blood respectively).

The direction of association was consistent for ER− breast cancer but the evidence of association was suggestive (OR per SD increase in expression level was 1.06 (95% CI 1.01, 1.12; P = 0.03) and OR per SD increase in expression level 1.19 (95% CI 0.99, 1.42; P = 0.06) in normal breast tissue and blood respectively). As we obtained only one SNP for the eQTL for *RHOD* from both breast tissue and blood, and none from breast cancer tissue, we were unable to perform multiple SNP sensitivity analyses.

There was some suggestive evidence that increased expression of *cell division cycle 42* (*CDC42)* was inversely associated with overall and ER+ breast cancer risk. (Overall breast cancer: observed OR per SD increase in expression level 0.91 (95% CI 0.84, 0.98; P = 0.02) and OR per SD increase in expression level 0.96 (95% CI 0.93, 1.00; P = 0.03) using eQTLs obtained from breast cancer tissue and blood in general population respectively. ER+ breast cancer: OR per SD increase in expression level 0.90 (95% CI 0.82, 0.99; P = 0.03) and 0.95 (95% CI 0.91, 0.98; P = 0.005) using eQTLs obtained from breast cancer tissue and blood, respectively). The direction of association was consistent for ER− breast cancer using eQTL from breast cancer but the evidence of association was weak (OR per SD increase in expression level 0.88 (95% CI 0.76, 1.02; P = 0.09)). Using an eQTL from blood, the evidence of association was inconclusive (OR per SD increase in expression level 1.01 (95% CI 0.95, 1.07; P = 0.78)).

For expression level of each gene, we calculated the power a priori, to detect an odds ratio (OR) of 1.2 (or conversely a protective OR of 0.80) given an alpha level of 0.05, the variance explained in the expression level of the gene by the SNP instrument and the sample size of the outcome dataset against overall, ER+ and ER− breast cancer risk, as described previously^18^. The power to detect the odds ratio of 1.2 (or equally 0.80) was > 99% for *RHOD* in overall, ER+ and ER− breast cancer using eQTL obtained from breast tissue and ≥ 30% using eQTL obtained from blood (Table 1). The power was > 99% for *CDC42* in overall, ER+ and ER− breast cancer using eQTL obtained from blood and ≥ 27.19 using eQTL obtained from breast cancer. The results for R^2^, F-statistic and power calculations are provided in Table 1.

**Figure 4:**
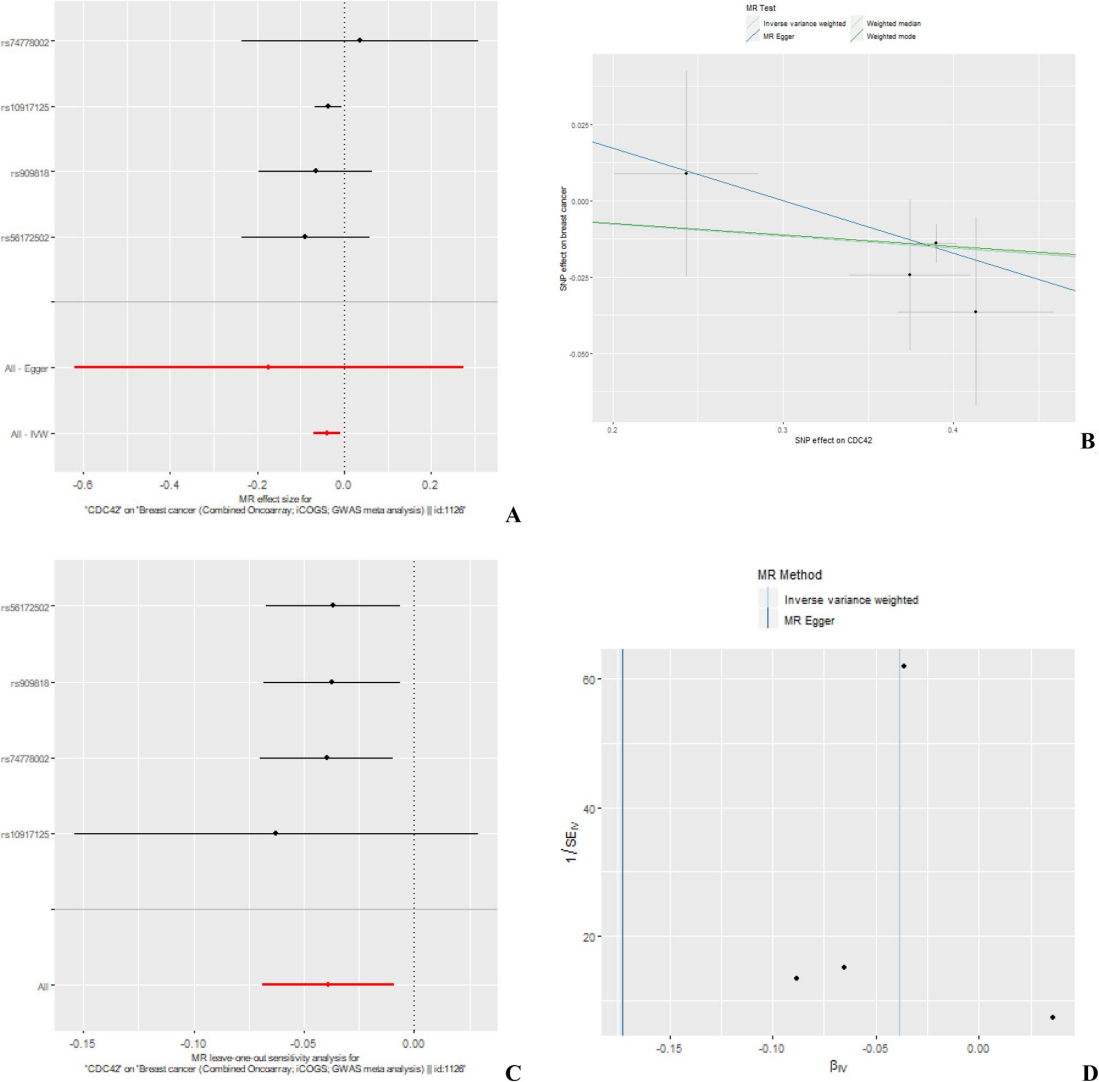
MR results for association between *CDC42* and overall breast cancer risk using eQTLs from blood. (**A**) Forest plot of single SNP analysis; (**B**) comparison of results using different MR methods; (**C**) leave-one-out sensitivity analysis; (**D**) funnel plot of IVW and MR-Egger regression.

**Figure 5:**
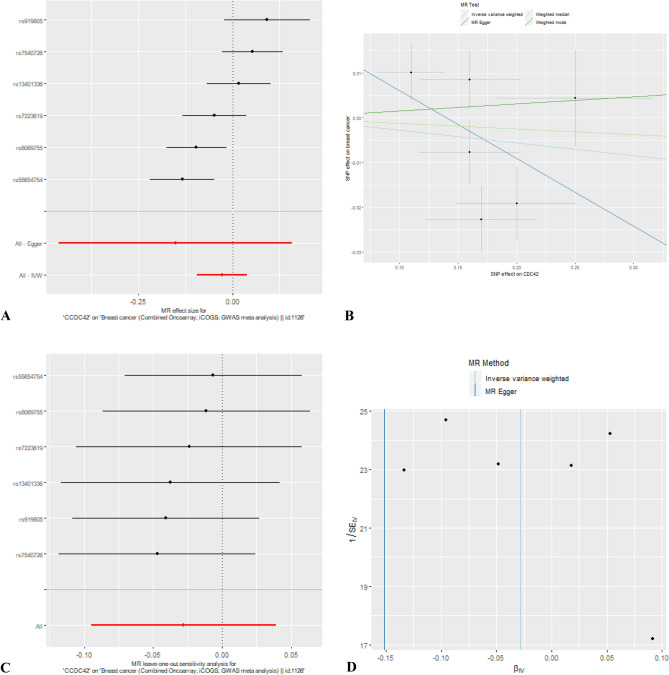
MR results for association between *CDC42* and overall breast cancer risk using eQTLs from breast cancer. (**A**) forest plot of single SNP analysis; (**B**) comparison of results using different MR methods; (**C**) leave-one-out sensitivity analysis; (**D**) funnel plot of IVW and MR-Egger regression.

In sensitivity analyses, using multiple SNPs obtained from breast cancer tissue, the direction of association for ER+ and ER− together was consistent with single SNP analyses but the evidence of association was weak (overall: OR per SD increase in expression level: 0.97; 95% CI 0.91, 1.04; P = 0.41, ER+ : OR per SD increase in expression level: 0.96; 95% CI 0.90, 1.03; P = 0.26 and ER−: OR per SD increase in expression level: 0.98; 95% CI 0.89, 1.08; P = 0.68). Using multiple SNPs obtained from blood, there was evidence of protective association with overall (OR per SD increase in expression level: 0.96; 95% CI 0.93, 0.99; P = 0.01) and ER+ breast cancer (OR per SD increase in expression level: 0.94; 95% CI 0.91, 0.98; P = 0.001) consistent to single SNP analyses. The evidence of association was inconclusive for ER− breast cancer risk (OR per SD increase in expression level: 1.01; 95% CI 0.96, 1.06; P = 0.66). For multiple SNP sensitivity analyses, the results for the effect of *CDC42* on overall and ER+ breast cancer were consistent across the various sensitivity analyses (Fig. 4 and 5). We did not find evidence of heterogeneity and pleiotropy across the individual causal effects (Supplementary Table 1).

In colocalization analyses for *RHOD* (using summary data from the GTEx platform) the posterior probability of colocalization (i.e. exposure and outcome are associated and share the same causal variant) was 84% for overall breast cancer risk and 98% for ER+ breast cancer risk suggesting that breast cancer risk and *RHOD* expression are both associated and share a single causal variant (Table 3). However, the evidence of colocalization was weak for ER− breast cancer risk and *RHOD* eQTLs (9%). There was weaker evidence of colocalization of the *CDC42* expression (using summary data from the eQTLGen platform) and breast cancer risk signals (Table 3).

**Table 3:** Results of colocalisation analyses for *RHOD* and *CDC42* genes.

	Overall breast cancer	ER+ breast cancer	ER− breast cancer
***RHOD (GTEx)***			
PP Hypothesis0	4.68 × 10^–10^	2.29 × 10^–11^	9.26 × 10^–9^
PP Hypothesis1	0.04	0.002	0.85
PP Hypothesis2	9.63 × 10^–10^	1.98 × 10^–10^	6.77 × 10^–10^
PP Hypothesis3	0.09	0.02	0.06
PP Hypothesis4	0.87	0.98	0.09
***CDC42 (eQTLGen)***			
PP Hypothesis0	9.36 × 10^–306^	1.78 × 10^–305^	2.54 × 10^–305^
PP Hypothesis1	0.33	0.62	0.89
PP Hypothesis2	3.42 × 10^–306^	3.15 × 10^–306^	3.05 × 10^–306^
PP Hypothesis3	0.12	0.11	0.11
PP Hypothesis4	0.55	0.27	0.007

*nsnp* number of SNPs used in the analysis, *PP* posterior probability.

**Figure 6:**
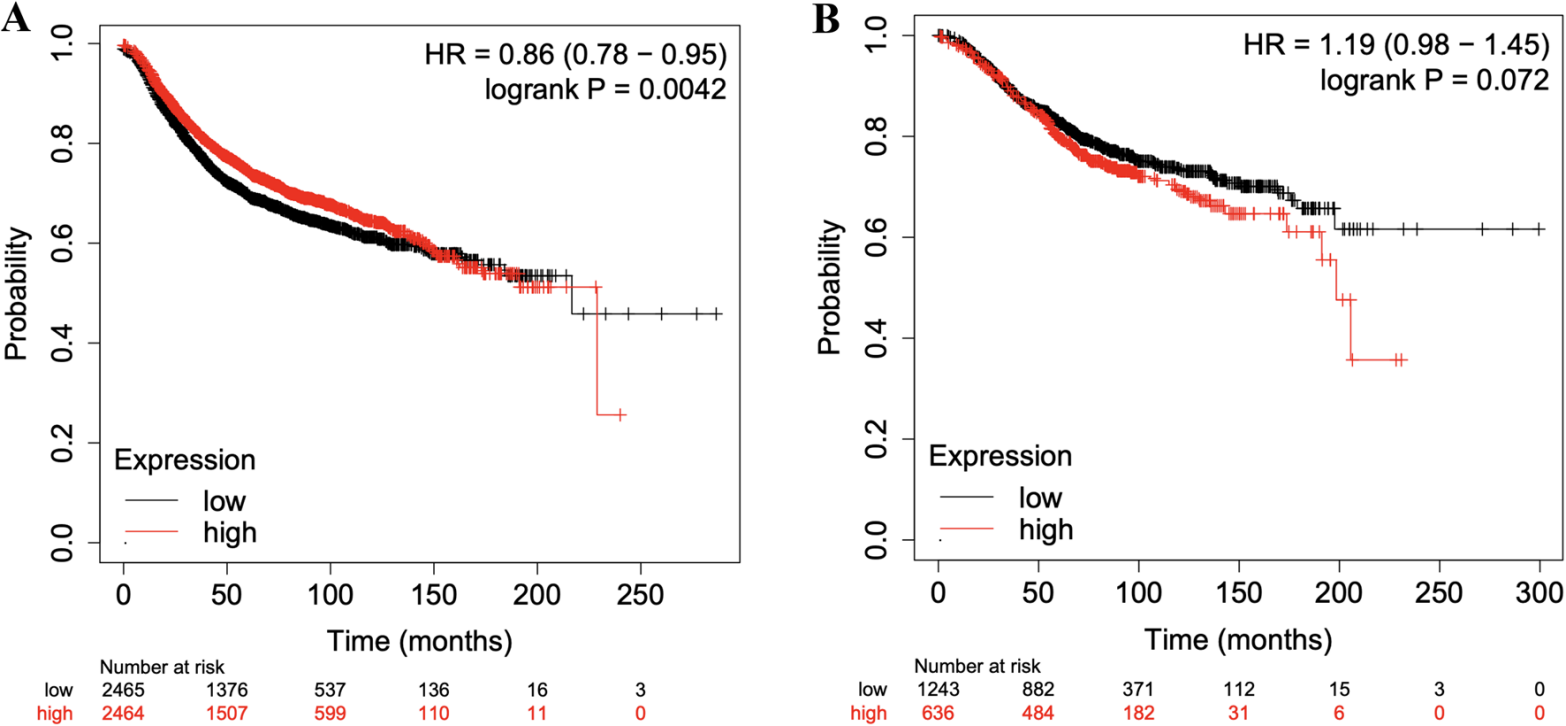
Kaplan–Meier plots of (**A**) recurrence-free survival in patients and (**B**) overall survival with ‘high’ and ‘low’ *RHOD* expression in overall breast cancer.

We then assessed the prognostic role of increased *RHOD* expression using KM Plotter^28^. Using the mean expression of the 2 *RHOD* probes (209885_at and 31846_at) within KM Plotter and splitting patients by an auto-select best cut off (and adjusted our P-values accordingly for multiple comparisons induced by the auto-selection procedure), we found that increased *RHOD* expression was associated with an improved Recurrence Free Survival (RFS) in overall breast cancer (Hazard Ratio (HR) 0.86, (95% CI 0.78, 0.95), P = 0.0042) but a worsening trend towards overall survival (HR 1.19, (95% CI 0.98, 1.45), P = 0.072) (Fig. 6). We repeated the same analyses above in the oestrogen receptor positive (ER+) subgroup, as our Mendelian Randomization results suggested a stronger relationship between *RHOD* and ER+ breast cancer. This repeat analysis showed a concordant result for RFS and OS, with higher *RHOD* expression showing a trend towards worsening PFS (HR 1.16 (95% CI 0.98–1.38), p = 0.08) and worse OS (HR 1.44 (95% CI 1.05–1.98) p = 0.025). We then assessed the relationship between PAM50 subtype, *RHOD* expression and RFS and OS, demonstrating conflicting results between OS and RFS. For increased *RHOD* expression the results were: Luminal A breast cancer, a protective effect on both OS (OS 0.52 (95% CI 0.33–0.81, p = 0.0034) and RFS 0.8, 95% CI 0.65–0.99, p = 0.039)); Luminal B a worse OS (HR 1.75 (95% CI 1.21–2.53, p = 0.0028) but a trend towards improved RFS (HR 0.85 (95% CI, 0.69–1.04, p = 0.11)); Her2 positive, a worse OS (HR 1.59 (95% CI, 1.06–2.37, p = 0.024) but improved PFS (0.86 (95% CI 0.68–1.1, p = 0.023)); and lastly Basal where there was a worse OS (HR 1.85 (95% CI, 1.27–2.71, p = 0.0013) and a trend towards worsening PFS (HR: 1.15 (95% CI 0.93–1.42, p = 0.21)). These results highlight that further work is needed in larger datasets or with functional laboratory experiments to investigate the underlying biology by manipulating *RHOD* in representative cell lines.

We then extracted the rates of amplifications and copy number alterations (CNA) in *RHOD* using cBioPortal^29^ and The Cancer Genome Atlas dataset^30^ and found a low percentage of amplification events across this dataset (47/993 patients, 4.7%). This varied by molecular subtype of breast cancer, ranging from 2.3% for basal-like breast cancer to 8.7% in the Luminal B (ER+ with aggressive features) subgroup (Table 4). For CNA, 26.7% of patients (266/933) had amplifications in *RHOD* (Table 5).

**Table 4:** Amplifications in *RHOD* in The Cancer Genome Atlas according to molecular subtype.

Molecular subtype	Number of patients	Number (percentage) with amplifications
Basal	130	3 (2.3)
Her2 positive	67	6 (9.0)
Luminal A	395	14 (3.5)
Luminal B	183	16 (8.7)
Normal	22	1 (4.5)
Unclassified	196	7 (3.6)
Overall	993	47 (4.7)

**Table 5:** Copy number alterations in *RHOD* in The Cancer Genome Atlas according to molecular subtype.

Molecular subtype	Number of patients	Number of copy number alterations (percentage)			
		-1 Loss	0 Neutral	1 Gain	2 Amplifcation
Basal	130	27 (20.8)	67 (51.5)	33 (25.4)	3 (2.3)
Her2 positive	67	11 (16.4)	30 (44.8)	20 (29.9)	6 (9.0)
Luminal A	395	44 (11.1)	264 (66.8)	73 (18.5)	14 (3.5)
Luminal B	183	37 (20.2)	76 (41.5)	54 (29.5)	16 (8.7)
Normal	22	1 (4.5)	17 (77.3)	3 (13.6)	1 (4.5)
Unclassified	196	27 (13.8)	127 (64.8)	36 (18.4)	7 (3.6)
Overall	993	147 (14.8)	581 (58.5)	219 (22.1)	47 (4.7)

To compare our novel results on *RHOD* expression with a known breast cancer-associated gene, we chose *TP53*, a well-known tumor suppressor^31^. Somatic mutations affecting the *TP53* gene (tumor protein p53) have been reported to be altered in breast cancer in approximately 20–40% of all cases, although this depends on the size of the tumor, molecular subtype and disease stage^32^. MR results showed evidence of a negative association between increased expression of *TP53* and ER+ breast cancer risk (OR per SD increase in expression level: 0.77; 95% CI 0.66, 0.90; P = 0.001) using eQTLs obtained from blood. The direction of effect was consistent with overall breast cancer risk, but the evidence was weak (OR per SD increase in expression level: 0.92; 95% CI 0.81, 1.05; P = 0.22). However, a positive association between *TP53* expression and ER− breast cancer was observed (OR per SD increase in expression level: 1.69; 95% CI 1.28, 2.24; P = 0.0002) consistent with recent literature (see “Discussion”)^17^.

## Discussion

Here we investigated the potential causal role of gene expression of Rho GTPases in breast cancer using a two-sample MR approach. Of the fourteen genes for which we found suitable germline genetic instruments, two—*RHOD* and *CDC42—*showed evidence of a link with breast cancer risk with the evidence being stronger for *RHOD*. The expression level of *RHOD* and *CDC42* demonstrated positive and inverse effects on overall breast cancer, respectively. Given that most cases in the overall breast cancer analysis were ER+, these associations were primarily driven by the ER+ subtype. There was a trend towards the same direction of effects in ER− breast cancer but this did not reach Bonferroni-corrected statistical significance, reflecting the lower numbers of ER− breast cancer cases in BCAC^11^.

To our knowledge, *RHOD* expression has not previously been implicated in breast cancer aetiology or indeed in any other cancer types, and thus our results are the first to link *RHOD* to cancer risk. There are very few studies on *RHOD* function compared to highly characterized Rho GTPases such as *CDC42*. RHOD was initially discovered as a regulator of membrane trafficking (reviewed in^33^) and contributes to the trafficking of tyrosine kinases such as SRC family and the PDGFRβ that are implicated in breast cancer progression^34–37^. It has also been reported to regulate several other cellular responses that are important for cellular transformation and early cancer development, including cell cycle progression, cytoskeletal dynamics, cell motility and centrosome duplication^38,39^.

**Figure 7:**
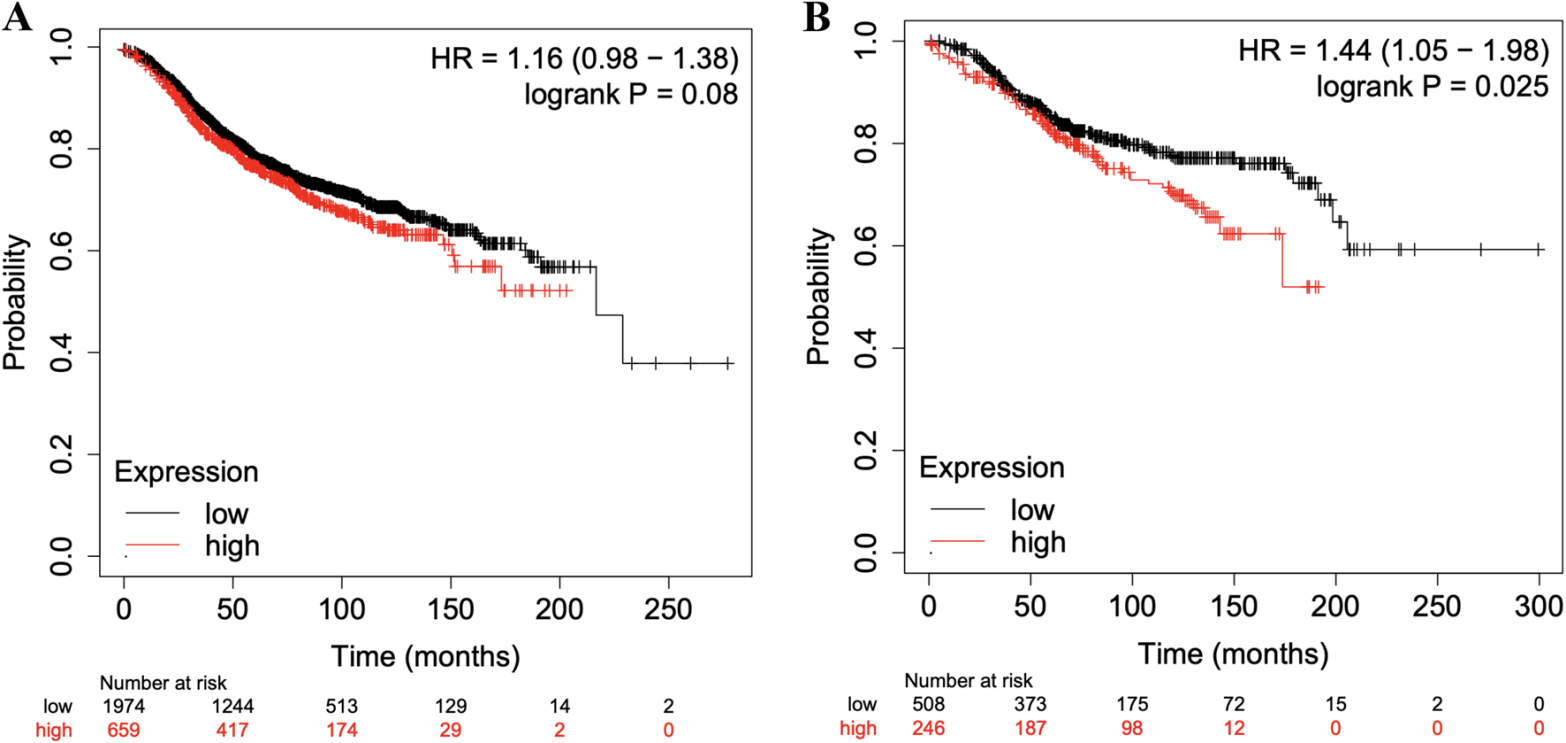
Kaplan–Meier plots of recurrence free survival (**A**) and overall survival (**B**) according to expression of *RHOD* in oestrogen receptor positive breast cancer by IHC.

Our results using publicly available datasets show a relatively low frequency of amplifications and copy number alterations within TGCA for *RHOD* (Tables 4 and 5) and a prognostic role of *RHOD* in both RFS and OS of oestrogen receptor positive breast cancer (Fig. 7). These data suggest that *RHOD* gene amplification could contribute to breast cancer progression in some women. Further analyses based on PAM50 subtype of breast cancer suggest that higher *RHOD* expression led to worse OS in more aggressive tumour subtypes (Luminal B, Her2 positive and Basal), but some results for RFS.were conflicting. There are numerous caveats and considerations that need to be taken into account when examining RFS and OS in this context—including the lack of detailed information as to the treatment that the patients included in these datasets received, the longer relapse times that we typically see in oestrogen receptor positive breast cancer (meaning that relationships often take many years to observe) and the fact that overall survival data assesses all causes of death including those unrelated to breast cancer.. Further work with larger numbers of women will be needed to assess whether the relationship between *RHOD* and RFS and OS is truly in opposite directions, with a focus on more aggressive breast cancer subtypes.

Our results demonstrated a protective effect of increased *TP53* expression on ER+ breast cancer risk, consistent with the known tumor suppressor role for *TP53*^31^. By contrast, the opposite effect was observed for ER− breast cancer. The relationship between germline and somatic changes affecting intra-tumoral *TP53* activity is complex and is altered dependent upon the rate of somatic mutations in *TP53* (approximately 80% in ER− and 20% in ER+ breast cancer). A discussion around this relationship and the effects of *TP53* on breast cancer risk has been recently published^17^ but interestingly the strength of effect on breast cancer risk we report with *RHOD* is even stronger than that of a well-established target (rs78378222 at *TP53*) on breast cancer risk.

Together, our results suggest that *RHOD* may play an important role in breast cancer aetiology and the mechanisms through which *RHOD* could drive breast cancer formation merit further investigation.

*CDC42* is a key regulator of both cell motility and cell cycle progression^40^ as well as in establishing normal epithelial cell polarity and promoting migratory polarity^7^. Compared to other Rho GTPases, *CDC42* is relatively less studied in cancer^41^. In single-cell in vitro experiments it promotes a mesenchymal phenotype with cellular invasion^42^ but its relationship with breast cancer phenotypes is complex. Previous reports have demonstrated overexpression in 42–57% of human breast cancers on a protein level associated with more aggressive clinical behaviour, ductal carcinomas and a poorer prognosis with increased cytoplasmic expression but the opposite with nuclear expression^43^. There are also previous reports suggesting that *CDC42* activation leads to a decreased migratory phenotype in breast cancer cell lines^44,45^. A genomic and transcriptomic analysis by the Molecular Taxonomy of the Breast Cancer International Consortium (METABRIC) demonstrated that less aggressive, ER+ tumours were enriched for altered expression of genes in the *CDC42* pathway^4^. Despite this, mutations in *CDC42-*related genes are very low at between 0.1 and 1.7% and the elevated *CDC42* expression in breast cancer is thought to be due to activation of oncogenes or cell surface receptors (for example; epidermal growth factor receptor (EGFR)) that lead to *CDC42* upregulation^46^. Differences in tissue-specific *CDC42* expression biasing the result are unlikely as the effects of SNPs for *CDC42* were derived from both breast cancer tissue and from blood and were in concordance. The more plausible explanation for the apparent protective effect of *CDC42* is that CDC42 maintains epithelial polarity^47,48^ and hence protects against cancer initiation. At later stages of breast cancer development, increased CDC42 expression could promote cancer progression via its effects on cell cycle progression and invasion^3^. The activity of most Rho GTPases, including RHOD and CDC42, is also controlled by over 70 guanine nucleotide exchange factors (GEFs), 60 GTPase-activating proteins (GAPs) and 3 Rho GDI proteins (guanine–nucleotide-dissociation inhibitors) that switch the Rho GTPases between active and inactive forms^39^. Interestingly, several GAPs are upregulated in basal-like breast cancers and contribute to breast cancer cell growth^49^.

We performed sensitivity analyses to disentangle the causal effects of gene expression from associations driven by horizontal pleiotropy, reverse causation, and genetic confounding through LD. We found robust evidence of colocalization for *RHOD*, suggesting that our MR findings could not be driven by genetic confounding through LD between eQTLs and other disease-causal SNPs strengthening the evidence of causality. Evidence of colocalization thus served as a complementary approach to reinforce the MR finding for *RHOD*.

We have tested the effects of Rho GTPases in human samples rather than cancer-derived cell lines or animal models that often give variable and inconsistent results. In contrast to conventional observational studies, MR is less susceptible to problems of confounding, reverse causation and measurement error. The use of two-sample MR enabled us to use the largest GWAS of breast cancer to date. Ideally, we would have liked to examine protein-level expression of the Rho GTPases as well as gene expression, but there are very few small studies on protein levels compared to the large datasets available for normal breast, breast cancer and blood-based transcriptomic profiles, and none to our knowledge for *RHOD*. Although we could not detect strong evidence of effects for Rho GTPases for the ER− breast cancer subtype, this could be due to a lack of power to detect smaller effect sizes in this cohort. Larger studies with matched germline genotype and tissue-specific normal and tumour gene expression will be required to improve the statistical power of similar MR analyses in the future and would help to causally link other members of the Rho GTPase pathway with breast cancer risk.

## Conclusion

We found robust evidence that *RHOD* expression may be causally and positively related to breast cancer risk, and that *CDC42* may potentially be causally and negatively related to breast cancer risk. Given that the activity of RHOD and CDC42 proteins is regulated by a variety of other proteins, it will be interesting to determine whether any of the genes encoding these regulators are also associated with breast cancer risk. The role of *RHOD* warrants further biological investigation to assess its role in breast carcinogenesis.

## Supplementary Information

Supplementary Table 1.

## Data Availability

All data analysed during this study were previously generated. Data availability repository links are given below: (1) BCAC GWAS: http://bcac.ccge.medschl.cam.ac.uk/bcacdata/oncoarray/oncoarray-and-combined-summary-result/gwas-summary-results-breast-cancer-risk-2017/. (2) TCGA eQTLs: https://albertlab.shinyapps.io/tcga_eqtl/. (3) GTEx: https://www.gtexportal.org/home/datasets.
